# *Leptotrichia* species isolated from a chronic recurrent corneal ulcer

**DOI:** 10.1016/j.ajoc.2021.101168

**Published:** 2021-07-22

**Authors:** Joanne W. Ho, Thomas Meirick, Dhruba J. SenGupta, Shu Feng

**Affiliations:** a Department of Ophthalmology, University of Washington. 325 9th Ave Box 359608, Seattle, WA, USA; b Department of Laboratory Medicine and Pathology, University of Washington Medical Center 1959 Pacific Street NE, Seattle, WA, USA

**Keywords:** Corneal ulcer, Keratitis, *Leptotrichia*, Persistent epithelial defect, Recurrent ulcer, Oropharyngeal flora

## Abstract

**Purpose:**

To report a case of recurrent corneal ulcer caused by an oropharyngeal cavity pathogen.

**Observations:**

A patient presented with recurrent corneal ulcers with hypopyon. *Leptotrichia* species was eventually isolated from the corneal ulcer on bacterial polymerase chain reaction (PCR) after many negative bacterial culture attempts. Due to correct identification of the pathogen, it was discovered that the patient was exposing her eye to saliva. Modification of patient behavior and initiation of the appropriate antibacterial treatment resulted in resolution of recurrent episodes of active infection.

**Conclusions:**

Although *Leptotrichia* species are not typically ocular pathogens, they can become pathogenic in the cornea with direct transmission from the oral cavity to the eye.

## 1 Introduction

Leptotrichia is a bacterial genus commonly present in the oral cavity. It can lead to tooth decay, and has more rarely been implicated in various other infectious processes throughout the body. However, Leptotrichia has never been reported to cause pathology within the eye. We present a case of a recurrent corneal ulcer found to be secondary to Leptotrichia, likely inoculated through poor contact lens hygiene.

### 1.1 Case report

A 56-year-old woman with history of recurrent corneal ulcers of the right eye was referred to an academic center for further workup and treatment. The patient had a history of recurrent right corneal erosions starting 2 years prior with minor trauma to her right eye from eyelash plucking. She did not have findings of epithelial basement membrane dystrophy in either eye. She first developed a corneal ulcer one year prior to presentation that was cultured with no growth on bacterial, fungal, or acanthamoeba cultures. It was treated with topical moxifloxacin and eventually healed with complete re-epithelization and resolution of the infiltrate. She continued using a bandage contact lens due to recurrent erosions and severe eye pain. This bandage contact lens was exchanged at least monthly and she remained on prophylactic topical tobramycin drops twice a day. A corneal ulcer recurred 3 months later and was treated again with topical moxifloxacin with eventual re-epithelization and resolution of the infiltrate. This ulcer was not cultured. Her vision at this point was 20/40 with a central stromal scar. She continued using a bandage contact lens and remained on topical moxifloxacin four times a day.

Two months later, she developed another corneal ulcer, now with deep stromal neovascularization and hypopyon. Due to the provider's concern for endophthalmitis, this was treated with vitreous tap and injection of intravitreal vancomycin and ceftazidime in addition to frequent topical fortified vancomycin and tobramycin drops. The vitreous sample yielded a negative fungal polymerase chain reaction (PCR) and bacterial culture. With continued topical antibiotic therapy, the hypopyon resolved and the infiltrate eventually consolidated into a scar.

Unfortunately, the hypopyon recurred twice in the next four months with worsening of the stromal infiltrate and development of an endothelial plaque. The hypopyon was treated each time with vitreous tap and injection of intravitreal vancomycin and ceftazidime and re-initiation of fortified vancomycin and tobramycin drops. Voriconazole drops were also prescribed due to concern for chronic fungal infection. Vitreous samples continued to be negative for bacterial culture and fungal PCR. Each period of intense treatment led to subsequent resolution of hypopyon and active infiltrate. Between episodes of active infection, she was continued on a bandage contact lens and topical moxifloxacin four times daily. Her vision declined to 20/400 due to dense stromal scarring.

At this time, she presented to our center for consideration of corneal biopsy with concern for an indolent fungal or acanthamoeba infection. She was noted to have a dense stromal scar with deep neovascularization, neovascularization of the iris, and posterior synechiae. She had no epithelial defect or signs of active infiltrate and her anterior chamber was quiet. It was felt that neovascularization and scar were likely the result of chronic inflammation caused by chronic recurrent ulcers. Given the recurrent nature of the keratitis despite topical antibiotics, there was consideration of possible herpetic etiology and she was started on prophylactic acyclovir. A corneal transplant was recommended, but the patient was now uninsured and did not have means to pursue surgical intervention. She opted to continue on a chronic bandage contact lens and moxifloxacin four times daily.

She returned 2 months later with worsening pain and irritation, now with hand motion vision. She was found to have an active central infiltrate with large epithelial deficit, 50 % focal thinning, dense neovascularization of the deep stroma, and hypopyon. Scrapings of the ulcer were plated for bacterial and fungal culture; additional scrapings were sent to the University of Washington Molecular Diagnostics Laboratory for bacterial, fungal, and viral PCR. She was started on fortified vancomycin and tobramycin drops.

Bacterial identification of the corneal scrapings was completed using broad-range bacterial 16S rRNA gene primers. The 16s rRNA gene was partially sequenced and the sequence was matched to Leptotrichia species in the Basic Local Alignment Search Tool (BLAST, NCBI) database. This method of bacterial detection by PCR is clinically available at select laboratories, including the University of Washington Molecular Diagnostics Laboratory. Fungal and acanthamoeba cultures, as well as fungal and viral PCR were negative. Bacterial cultures grew two colonies of coagulase negative staphylococcus, which was thought to be a contaminant. The bandage contact lens was discontinued and she was transitioned to fortified tobramycin and cefazolin drops with continued improvement of the ulcer and resolution of the hypopyon. The visual acuity at this time was hand motion.

*Leptotrichia* species are anaerobic Gram-negative rods that are constituents of normal oral flora. When this was conveyed to the patient, she admitted that she regularly licked her finger to wet her lower eyelid when she felt that her contact lens was irritating. Given chronic recurrent infections despite periods of relative inactivity and negative cultures prior to this, it was suspected that her recurrences were related to re-exposure to mouth flora with chronic bandage contact lens use. She was strongly encouraged to discontinue this habit and stopped her bandage contact lens use. The patient did not have any recurrences of infection after 1 year of follow-up.

## 2 Discussion

*Leptotrichia* was established as a genus in 1879 to classify specific filamentous microorganisms in the oral cavity.^1^ The *Leptotrichia* species is characterized by Gram-negative non-sporing, non-motile, anaerobic, saccharolytic rods. Lactic acid is a major metabolic end product of *Leptotrichia*, which may contribute to tooth decay.^2^

In addition to the oral cavity, *Leptotrichia* is found in the intestine and human female genitalia. It has been detected orally in up to 71 % of children after tooth eruption and reportedly colonizes implants of edentulous patients.^1^^,^^2^ While typically not considered pathogenic, *Leptotrichia* infections have been reported in a variety of human infections including gingivitis, periodontitis, acute appendicitis, bacterial vaginosis, aortic aneurysms, chancroids, salpingitis, and cellulitis.^1^^,^^2^ Of note, *Leptotrichia* has also been isolated in peritoneal fluid and blood from immunocompromised patients such as those with neutropenia and HIV.^1^^,^^3^^,^^4^

Identifying *Leptotrichia* can be difficult as fresh cells may stain Gram-positive when cultured. In addition, some strains are strictly anaerobic or facultative anaerobic, while others grow best with carbon dioxide. It is now recommended to use 16S rRNA gene identification as a more reliable and feasible identification method for *Leptotrichia*.^2^ In our case, while the culprit causing the initial corneal ulcer was likely not *Leptotrichia*, its identification by bacterial PCR uncovered the causative agent of the patient's recurrent episode and the fact that the patient was exposing oral microorganisms to her eye. This diagnosis was helpful in guiding antibiotic choice, and crucial in appropriately modifying patient behavior.

*Leptotrichia* species are generally susceptible to penicillins, cephalosporins, clindamycin, metronidazole, rifampin, tetracyclines, imipenem, and chloramphenicol.^2^ Of note, some strains have developed resistance to many commonly used topical ophthalmic antibiotics including erythromycin, vancomycin, tobramycin, and fluoroquinolones.^2^ In our patient, the causative Leptotrichia strain introduced by mouth flora may have become resistant to fluoroquinolones, leading to recurrence of the ulcer despite chronic topical moxifloxacin use. The ulcer was finally adequately treated with fortified tobramycin and cefazolin and discontinuation of contact lens.

Direct inoculation of the cornea presumably from the oral cavity has been reported. Such cases have identified *Rothia dentocaiosa*, *Kingella species*, and *Eikenella corrodens*.^5^, ^6^, ^7^, ^8^ To our knowledge, this case is the first confirmation of *Leptotrichia* species causing a corneal ulcer.

## 3 Conclusions

When considering etiology of recurrent or recalcitrant corneal ulcers, one should consider the possibility of oral flora in addition to fungal and viral microorganisms. In this case, the patient's chronic bandage contact lens use and ocular hygiene habits resulted in repeat inoculation of the cornea by oropharyngeal flora leading to a chronic, recurrent corneal ulcer with subsequent severe vision loss.

## Patient consent

The patient consented to publication of the case in writing.

## Acknowledgements and disclosures

Funding: This study was supported by 10.13039/100001818Research to Prevent Blindness. The funding organization had no role in the design or conduct of this research.

## Authorship

All authors attest that they meet the current ICMJE criteria for Authorship.

## Declaration of competing interest

The following authors have no financial disclosures: JWH, TM, DS, SF.
